# Care of the Elderly—The Way Ahead

**Published:** 1985-07

**Authors:** G. K. Wilcock

**Affiliations:** Professor in Care of the Elderly, University of Bristol


### Care of the Elderly—The Way Ahead?

**G. K. Wilcock, B.Sc., D.M.(Oxon), F.R.C.P.**
Professor in Care of the Elderly, University of Bristol

### INTRODUCTION

Three main factors have influenced the theme which I shall be developing in this paper. The first and most important is the need to provide as high a standard of care as possible, for as many elderly people as possible. The second is the necessity to adopt a practical approach in light of the demographic changes which are occurring in association with both a relative and an absolute shortage of resources. The third factor is the experience gained during the past 10 years whilst working with several different patterns of health care delivery to the elderly.

Table 1 shows the changes occurring within different age bands in people aged 65 and over, between 1978 and the end of this century. Although the number of our elders will have risen slightly, the major changes will occur in those aged 75 and over. It is generally agreed that a person's 75th birthday marks a watershed in his or her life, since for many there is subsequently an increasing likelihood of acquiring degenerative conditions which may result in some loss of independence. It is this age group that makes particularly heavy demands upon health and other caring services. It can also be seen from the Table that a small drop in the number of people aged 65–74 years of age will occur. Many people in this age group are heavily involved in caring for more elderly persons, either because they are the sons and daughters of people in their 80's, or because they play a major role in one of the many voluntary bodies concerned with providing care for our elderly.

In practical terms it has been calculated that about a quarter of medical beds and three-quarters of geriatric beds are already occupied by people aged 75 and over,[1] and according to Irvine,[2] Livesley has extrapolated this trend to show that by the end of the century, 90% of all acute beds, not just acute general medical and geriatric beds, will be occupied by people over the age of 65, and 70% by people aged 75 and over, unless there is a change in the approach to care of the elderly. Although there is the contrary view that antemortem pathology will be compressed into an ever decreasing period by advances in medicine, necessitating less health care, this is unlikely as expenses of the current trend emphasises the need for both a flexible approach to the pattern of delivery of services to our elders, as well as an increasing need for resources. These needs go hand in hand.

**Table 1**

**Projected population aged 65 and over in Great Britain, 1978–2001. (From OPCS Population Projections 1972–2017, 1978 = 100)**

| | *65–74* | *75–84* | *85+* | *65+* |
|---|---|---|---|---|
| 1978 (thous) | 5022 | 2393 | 527 | 7942 |
| 1981 (%) | 99.6 | 107.2 | 105.9 | 102.3 |
| 1991 (%) | 95.3 | 117.3 | 140.0 | 104.9 |
| 2001 (%) | 86.9 | 115.7 | 160.7 | 100.5 |

Lecture delivered to the Bristol Medico-Chirurgical Society, 13th February 1985.

### ORGANISATION OF HOSPITAL CARE FOR THE ELDERLY

### (i) GERIATRIC AND GENERAL MEDICINE

Broadly speaking, departments of geriatric medicine practice one of four patterns of delivery of care. The original custodial services for the chronically sick elderly person have largely given way to the development of separate autonomous departments of geriatric medicine. These usually have a finite number of beds, of which hopefully today at least a reasonable proportion are provided on the main District General Hospital (DGH) site. They admit as many patients as possible and if adequate DGH-type facilities are available, can provide an excellent service with a strong acute element and a first rate standard of care. However, the finite number of beds usually results in a spill-over of patients into other departments and they are deprived of the specialist approach. In addition, in this model only staff working on the unit, whether in a permanent or rotating position, actually experience the modern approach to caring for the elderly.

The third pattern is essentially similar to the separate autonomous service, but with an admission policy that attempts to take in all patients over a certain age, commonly 75 and over. To work well this needs a large number of DGH beds, or again there will be an overspill of elderly patients into the beds of other services. Although an effective service usually results, and this approach is favoured by many, it does not have the advantages of a fully integrated service. In the latter, although different patterns exist, there is usually one set of acute wards for medical patients of all ages, with the consultant physicians arranged as 'firms', one member of each firm having special responsibility for the elderly. He will have been trained in general as well as geriatric medicine, goes on general medical take alongside his medical colleague(s), and runs the rehabilitation and continuing care wards for his firm, as well as providing a service for those elderly patients not admitted as medical 'emergencies'. The junior staff the majority of whom rotate, are shared by all the physicians and for many of the juniors this will also include a spell attached to a rehabilitation ward.

Evaluation of an integrated approach has been undertaken in Newcastle[3] where it was shown that although the inclusion of the acutely ill elderly patients on the general admission wards resulted in the average age of the patients being older than in other acute medical units, the mean length of stay for all patients on the acute ward fell. This was not the result of an increased transfer of patients to rehabilitation or back-up wards, since only a small proportion were transferred. This efficient usage of medical admission beds was almost certainly contributed to by many different factors. These include the availability of DGH facilities for all acutely ill elderly patients, but also the equally rapid availability for all elderly patients, irrespective of the consultant under whom they were admitted, of advice from the consultant with responsibility for care of the elderly, and access to his resources. Probably more important, however, was the training of the junior staff, nurses and remedial therapists attached to the 'firm'. They would have applied the same approach to treatment to all elderly patients under the care of their 'firm'. There are other advantages to an integrated approach, and these include: the physician with special responsibility for care of the elderly having a personal involvement in, and commitment to, the efficient functioning of the acute unit; medical, nursing and other staff on the unit become personally responsible for the discharge of their elderly patients rather than regarding them as waiting for transfer to a geriatric unit; and very importantly it allows those caring for the elderly to work alongside their colleagues in other specialties, fostering closer professional relationships with greater mutual understanding of each other's roles than is possible when the geriatric department functions in a separate unit.

Integration results in an extremely efficient use of DGH beds and a much larger pool of health service personnel in all the relevant disciplines are being trained in the modern approach to care of the elderly, taking the knowledge and the experience gained with them when they move to other areas. This last point is particularly important, since although we all try and teach undergraduate and postgraduate students the correct approach to caring for elderly patients, medicine is best learnt by serving an apprenticeship. Just as it is considered important for most SHO's and registrars on the 'medical' side to gain experience in general medicine, it is now equally essential for them to have had some training in geriatric medicine, and this cannot always be provided by establishing rotating appointments, since in many instances it would not be possible for a rotational scheme to include all or even the majority of the junior staff.

In an ideal world it might seem that the best way of tackling the increasing number of older and frailer patients would be to expand the number of consultant geriatricians, and of course the resources with which they work, but this is clearly impractical. At the moment there are around 500 physicians in geriatric medicine in Great Britain, which is a long way short of the number needed even if the guideline of one consultant geriatrician per 10,000 people aged 65 and over is scaled down. Much of this is a recruitment problem, and if another hundred posts became available tomorrow, I doubt if we could find enough adequately trained applicants to fill them. Although there is some easement, it is proving difficult to attract high calibre applicants to many traditional senior registrar and consultant posts in units practising solely geriatric medicine. On the other hand, evidence is emerging that joint training and consultant posts are an increasing attraction to many doctors already training in different branches of medicine who are prepared to redirect their training.

Much scepticism has surrounded the evolution of joint appointments, and some of this is concentrated upon the worry that a consultant with this dual responsibility will neglect his elderly charges. This is not the case as those in joint appointments will all have been specially trained in geriatric as well as general medicine, and have care of the elderly as the major part of their job description and contractual obligation.

Another area of concern is the creation of a joint appointment alongside an existing whole-time geriatrician who has not been given the option to change to a joint appointment or who, as is more usually the case, does not wish to. Very reasonable fears about the development of lower status for the consultant in the older style appointment, and about the possibility of his newer colleague having a smaller workload are not in practice realised.

### (ii) OTHER SERVICES

There are many other areas within hospital services where a good case can be made for integration with the geriatric department. Those that spring most readily to mind include orthopaedics/trauma, urology, care of the patient who has had a cerebrovascular accident, and psychogeriatrics where I believe the whole approach needs re-thinking. Space precludes further mention of more than one of these, and the Hastings geriatric/orthopaedic ward is a good example of the benefits of such integration.[4] They have shown that this approach significantly reduces the length of stay of patients operated on for a fractured neck of femur, both in the orthopaedic ward and in terms of the total duration of hospital stay.

### COMMUNITY CARE

We should all support the concept of community care, since the community is where the majority of patients would like to live, but this approach is only practical so long as there are adequate resources to back it up and as long as it does not result in inappropriate discharges to a community that cannot reallly cope. The present trend of increasing the number of dependent people in the community is causing more stress than is often realised, usually because of inadequate support for the carers. We already have the position where the provision of home helps and meals on wheels is falling behind the demographic trends, and there has been, relatively speaking, a fall in the provision of local authority residential accommodation in recent years. This is resulting in a rebound effect which, coupled with the increasing numbers of frailer people, is changing the face of hospital continuing care. The community is now expected to care for many of those who a few years ago would have been in hospital. Their hospital beds are being filled from the ever increasing pool of extremely dependent people, and many of those we are discharging or not admitting will eventually need hospital care when their dependency level increases. Many continuing care wards have staffing levels determined some years ago and, even if not subjected to recent cutbacks, are now inadequately staffed to cope with an ever growing number of increasingly frailer and more dependent patients.

The escalating dependency load is further compounded by a rise in the number of demented patients who find their way onto wards where the nurses have not been properly trained to manage either the patients, or their own reactions to them. This results partly from the rise in the number of people with dementia, but also from the trend to nurse them in the community for longer, such that by the time they require institutionalisation they meet the criteria for geriatric rather than psychogeriatric care. Previously many of them would have been admitted earlier in their lives to psychogeriatric wards, usually while still ambulant, and have stayed there for their allotted span. If these patients are to be cared for in the community for longer, then we should train our staff appropriately and provide adequate resources both outside the hospital and within continuing care wards to meet the needs that arise.

The mushrooming of private residential and nursing homes should be considered an additional resource, but are also a cause for concern. Many elderly people are admitted to one of these establishments without an adequate assessment. In addition, quite naturally the proprietors reserve the right to choose whom they will admit and whom they will discharge. Again this means that the more socially difficult and increasingly dependent residents will add to the difficulties experienced by those responsible for Health Service continuing care wards. The value of a medical assessment before admission to a social services welfare home has been demonstrated by Brocklehurst,[5] and the same principle should apply to the private sector. There is clearly an important role here for departments of geriatric medicine, but how assessment could be best achieved and the resources needed requires further consideration.

The contribution of the voluntary organisations in relation to the statutory services needs clarification, and I believe that they should have a say in determining their role. Health Authorities and Social Services departments should encourage us all to make our professional expertise more readily available, and considerable potential exists for greater cooperation.

### IN CONCLUSION

There are many other areas of concern, particularly to those of us with a special responsibility for caring for the elderly, including community hospitals and general practitioner screening. Had space been available to explore these, I am certain that the theme would have been the same, namely that of the need for greater integration. There is an increasing need to regard departments of geriatric medicine as a source for others to call upon, not just for support, but also for education. On its own, politically unpopular though it may be, this approach will not be enough and some increase in resources is essential to help match the consequences of the demographic changes described earlier. The adoption of a flexible approach to the development of patterns of care, however, may result in the overall use of less resources, both in the hospital and in the community.

### REFERENCES

1. DHSS (1981) The Respective Roles of the General Acute and Geriatric Sectors in the Care of the Elderly Hospital In-patient. London, HMSO.

2. IRVINE, R. E. (1984) Geriatric medicine and general internal medicine. *Journal of the Royal College of Physicians of London* **18**, 21–24.
3. EVANS, J. G. (1983) Integration of geriatric with general medical services in Newcastle. *Lancet* **i**, 1430–1433.
4. IRVINE, R. E. (1982) Geriatric orthopaedic unit. In Coakley D. (ed.). Establishing a Geriatric Service. London, Croom Helm.
5. BROCKLEHURST, J. C. et al. (1978) Medical screening of old people accepted for residential care. *Lancet* **ii**, 141–142.

